# Permanent tooth avulsions: A retrospective analysis of the demographics and aetiology of cases at a tertiary hospital in Sydney, Australia

**DOI:** 10.1111/aej.12891

**Published:** 2024-09-19

**Authors:** Julia Bradshaw, Bill Kahler, Shanika Nanayakkara, Neeta Prabhu

**Affiliations:** ^1^ School of Dentistry, Faculty of Medicine and Health The University of Sydney Sydney New South Wales Australia; ^2^ Department of Paediatric Dentistry Westmead Centre for Oral Health Westmead New South Wales Australia

**Keywords:** children, dental trauma, epidemiology, tooth avulsion, tooth replantation

## Abstract

This retrospective analysis reviewed the demographics of patients sustaining dental avulsion injuries at a tertiary dental hospital in Sydney, Australia. Data were extracted from dental records of patients who presented with avulsed permanent anterior teeth and were treated between 1 January 2001 and 30 June 2021. Demographic, clinical and radiographic data from 91 patients with 117 avulsed permanent anterior teeth were available for analysis. The median age of the patients was 12 years (IQR 9.0–17.0). Males accounted for 68.4% of avulsion injuries. Non‐organised sports were the most common cause of injury (42.7%). Maxillary central incisors were the most frequently avulsed tooth (83.3%). Peak prevalence of injuries occurred on the weekend. The findings from this study may reflect regional factors such as climate and participation in sport. Anticipatory guidance should be provided to patients at elevated risk of dental avulsion.

## INTRODUCTION

Traumatic dental injuries (TDIs) present a significant global public health concern. Epidemiological studies have demonstrated an annual incidence of TDIs of up to 4.5% [1]. Dental trauma peaks in early life and adolescence, both important periods of psychological development [2, 3]. Based on the available evidence, maxillary central incisors are the most commonly injured tooth [2]. Previous studies report that males experience a higher prevalence of dental trauma in the permanent dentition than females; however, this trend appears to be on the decline, possibly reflecting increased female participation in sport [2]. Falls, sport, violence and road traffic accidents are commonly cited causes of dental trauma [4]. Risk factors for TDIs include increased overjet and inadequate lip coverage [4, 5]. Other factors identified in the literature include risk‐taking children, children being bullied, children with attention deficit hyperactivity disorder (ADHD), learning difficulties, physical limitations, obesity and oral piercings [4].

Tooth avulsion is defined as the complete exarticulation of a tooth from its socket. Avulsion injuries are one of the most serious TDIs, resulting in damage to the neurovascular supply of the tooth, the periodontal ligament (PDL) and supporting gingival tissues and the alveolar bone. Avulsion injuries account for 0.5%–16% of TDIs in the permanent dentition [6, 7, 8]. Avulsion injuries peak in children aged 7–9 years old, likely reflecting the loosely structured PDL in recently erupted teeth and less mineralised alveolar bone [6, 9, 10]. Replantation of avulsed permanent teeth is generally accepted as the best practice; however, it is associated with significant pulpal and periodontal complications such as pulp necrosis, inflammatory external root resorption and replacement external root resorption [9, 10]. Since 2001, the International Association of Dental Traumatology has published multiple evidence‐based guidelines on the recommended management of TDIs, attempting to standardise management. Management of avulsed permanent teeth is influenced by the root development of the injured tooth and the extra‐oral dry time [11]. Overall, functional health is observed between 16% and 58% of avulsed permanent teeth [9, 12, 13, 14, 15, 16, 17].

There have been no publications to date in Australasia regarding outcomes or survival of replanted permanent avulsed teeth. This publication aims to review the demographics of patients who had ongoing treatment for avulsion injuries at a tertiary hospital in Sydney, Australia.

## MATERIALS AND METHODS

**FIGURE 1:**
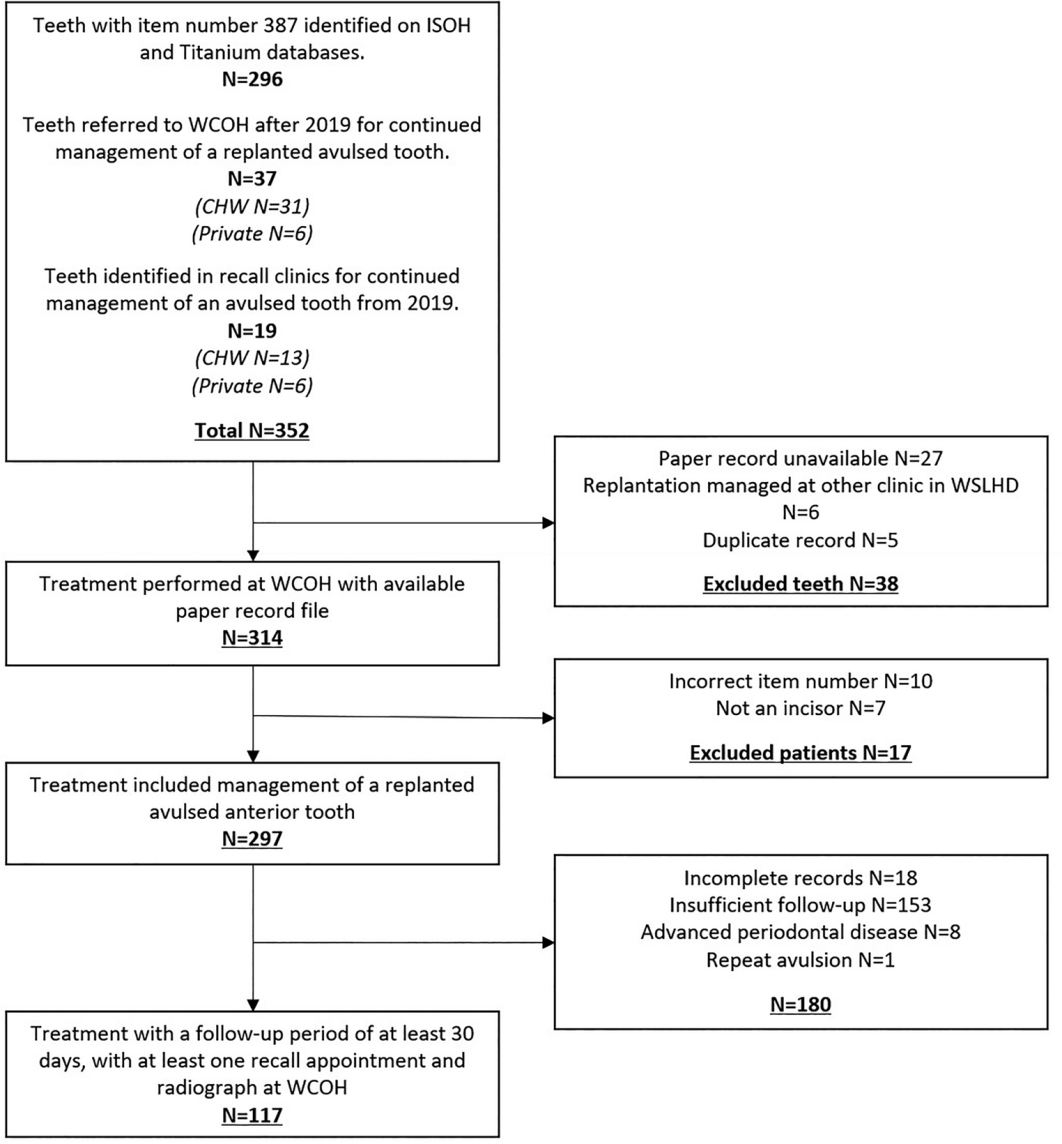
Flow chart—inclusion and exclusion criteria used to determine the study population in this research.

Ethics approval for the study was obtained from the Western Sydney Local Health District (WSLHD) (HREC reference: 2007‐05 QA). This retrospective study involved the extraction of data from dental records of patients with ongoing treatment at the Westmead Centre for Oral Health (WCOH), Sydney, Australia between January 2001 and June 2021. Included patients had one or more avulsed and replanted permanent anterior teeth, which may have been replanted at WCOH or referred to WCOH for ongoing management after the initial emergency replantation. Referral locations included the Children's Hospital at Westmead (CHW) and private practice. Inclusion criteria included [1] traumatised permanent anterior tooth with an avulsion injury (complete displacement of a tooth from its socket), [2] the avulsed tooth was replanted, [3] the tooth was followed up at WCOH for a minimum of 30 days (unless the tooth was lost prior to this time) and [4] the follow‐up appointment had an accompanying radiograph. The inclusion and exclusion criteria are outlined in Figure 1. All stages of root development were included. Stage of root development was divided into six stages according to the Moorrees classification of root development [18]. Teeth with Stages 1–4 root development were considered immature, while teeth with Stage 5 and 6 development were considered mature. Data obtained from records included patient demographics, trauma history, treatment provided and follow‐up information. Oral predisposing factors were excluded. Number of visits in the first 12 months was recorded for patients who attended Westmead Hospital/WCOH for the initial replantation of the avulsed tooth. WCOH is part of a larger Westmead Hospital and shares mutual records. Appointments were excluded if they were unrelated to the dental trauma.

The data collected were entered manually into an MS Excel spreadsheet (Microsoft Office 365 Excel) and the statistical analysis was performed using IBM SPSS Statistics, version 23 (IBM SPSS Inc., Chicago, IL, USA). The level of significance for all statistical analyses was set at ≤0.05. Following normality assessment, quantitative data were summarised using median and percentiles while categorical data were summarised using percentages. Non‐parametric Mann–Whitney *U* test was used to compare means between groups and Spearman's rank correlation coefficient to assess correlation between variables.

## RESULTS

The study population consisted of 91 patients with 117 avulsed teeth. The median follow‐up time was 18.5 months (median 556 days, IQR 250–963 days) (Table 1). The median number of visits to WCOH was seven in the first 12 months following the tooth avulsion injury (Table 1). There was no significant difference in the number of visits between mature and immature avulsions (*p* = 0.48). Further, no significant correlation was observed between age of the participants and number of visits (*r* = −0.16, *p* = 0.21).

**TABLE 1:** Observation time and visiting patterns.

Observation time (days) (*N* = 117)	
Min	14
Max	4418
Median (25th–75th percentile)	556 (250–963)
Number of visits (*N* = 56)	
Min	2
Max	20
Median (25th–75th percentile)	7.0 (5.0–9.0)
Immature (*N* = 6)	
Min	5
Max	10
Median (25th–75th percentile)	5.5 (5.0–6.8)
Mature (*N* = 50)	
Min	2
Max	20
Median (25th–75th percentile)	7.0 (6.0–9.0)

Age of the patients ranged between 5 and 90 years, with a median age of 12 years (IQR 9.0–17.0) (Table 2). Majority of the study population were males accounting for 68.4% of avulsion injures, while females accounted for 31.6% (Table 2). Thirteen patients (11.1%) were categorised as having a significant medical history (Table 2) including autism, epilepsy, ADHD, von Willebrand disease, juvenile arthritis, Type 1 diabetes mellitus, osteoporosis (including patients on bisphosphonates), bipolar disorder and cerebral palsy. A majority (70.1%) of patients presented to a hospital emergency department after an avulsion injury, while 18.8% presented directly to WCOH, 8.5% presented to a private general dental practitioner (GDP) and 1.7% presented to a general medical practitioner (GMP) (Table 2).

**TABLE 2:** Distribution of patient demographics and clinical characteristics of the avulsed teeth (*N* = 117).

	*N* (%)
Age	
Min	5 (years)
Max	90 (years)
Median (25th–75th percentile)	12.0 (9.0–17.0) (years)
Gender	
Male	80 (68.4%)
Female	37 (31.6%)
Significant medical history	
Yes	13 (11.1%)
No	104 (88.9%)
Cause of injury	
Assault	14 (12.0%)
Dog bite	1 (0.9%)
Fall or collision	36 (30.8%)
Non‐organised sport	50 (42.7%)
Organised sport	13 (11.1%)
Seizure	2 (1.7%)
Missing data	1 (0.9%)
Location of initial presentation	
Public dental hospital	22 (18.8%)
Emergency department	82 (70.1%)
Private GDP	10 (8.5%)
GMP	2 (1.7%)
Missing data	1 (0.9%)
Location of avulsion management	
Westmead Hospital (including WCOH)	65 (55.6%)
CHW	41 (35.0%)
Private	9 (7.7%)
Other	2 (1.7%)

**FIGURE 2:**
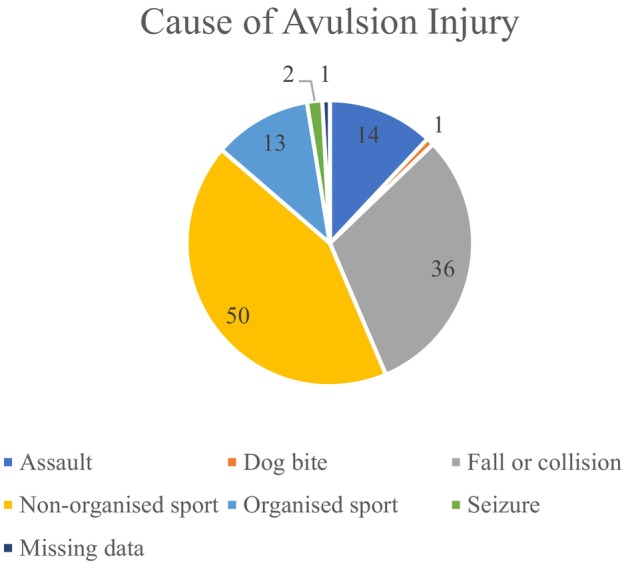
Cause of avulsion injury (*N* = 117).

Non‐organised sport (such as scooters, push bikes, trampolines and swimming pools) accounted for the most common cause of injury in this cohort (42.7%), followed by falls or collisions (30.8%) (Table 2, Figure 2). Organised sport was defined as sporting activity affiliated with a school or club (such as rugby, cricket and karate) and accounted for 11.1% of injuries (Table 2).

**FIGURE 3:**
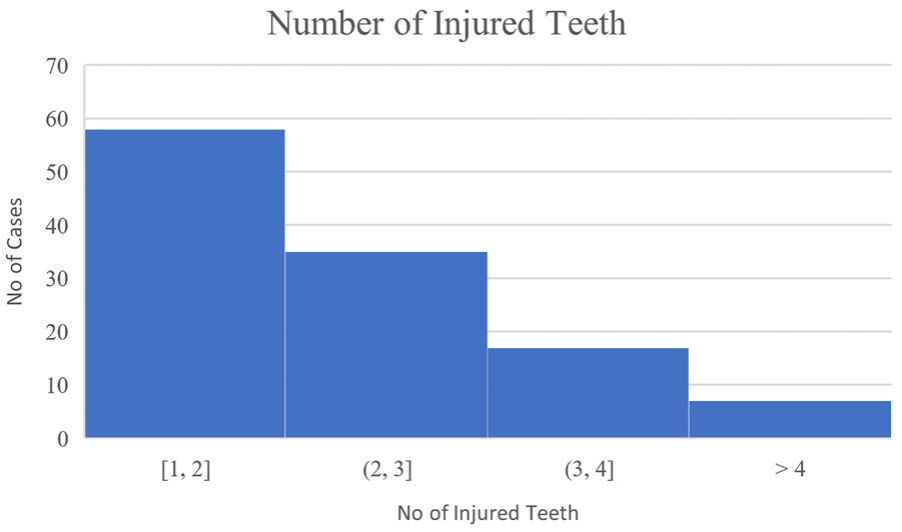
Total number of injured teeth (including avulsed tooth) (*N* = 117).

Concomitant extra‐oral injuries such as facial lacerations and bony fractures had a prevalence of 43.6% (Table 3). Gingival lacerations were observed in 44.4% of teeth, while 18.8% recorded an alveolar fracture (Table 3). The median number of injured teeth (including the avulsed tooth) was three (Table 3, Figure 3). Maxillary central incisors were the most frequently avulsed tooth (83.8%) (Table 4). Mandibular teeth accounted for 6.8% of injuries (Table 4). Concomitant crown fractures were observed in 12.0% of teeth, all of which were mature (Table 4). A minority of teeth in the data were immature (15.3%) (Table 4). A minority of teeth were categorised as compromised (5.1%), all of which were mature (Table 4). Compromised teeth included those with early periodontal disease (*N* = 2), large restorations (*N* = 3) and previous trauma (*N* = 1). Two teeth included in the study had concomitant apical root fractures (Table 3).

**TABLE 3:** Descriptive statistics for concomitant injuries (*N* = 117).

	*N* (%)
Total number of injured teeth	
Min (no. of teeth)	1
Max (no. of teeth)	9
Median (25th–75th percentile) (no. of teeth)	3 (2.0–3.0)
Extra‐oral injury	
Yes	51 (43.6%)
No	66 (56.4%)
Apical root fracture	
Yes	2 (1.7%)
No	115 (98.3%)
Alveolar fracture	
Yes	22 (18.8%)
No	95 (81.2%)
Gingival laceration	
Yes	52 (44.4%)
No	65 (55.6%)

**TABLE 4:** Clinical characteristics of the avulsed teeth (*N* = 117).

	*N* (%)
Tooth location	
Maxilla	109 (93.2%)
Central incisors	98 (83.8%)
Tooth 11	50 (42.7%)
Tooth 21	48 (41.0%)
Lateral incisor	11 (9.4%)
Tooth 12	8 (6.8%)
Tooth 22	3 (2.6%)
Mandible	8 (6.8%)
Central incisor	5 (4.3%)
Tooth 31	3 (2.6%)
Tooth 41	2 (1.7%)
Lateral incisor	3 (2.6%)
Tooth 32	2 (1.7%)
Tooth 42	1 (0.9%)
Crown fracture	
Yes	14 (12.0%)
No	103 (88.0%)
Root development	
Mature	99 (84.6%)
Immature	18 (15.4%)
Stage of root development	
1	0 (0.0%)
2	8 (6.8%)
3	3 (2.6%)
4	7 (6.0%)
5	12 (10.3%)
6	87 (74.4%)
Compromised tooth	
Yes	6 (5.1%)
No	111 (94.9%)

The highest prevalence of traumas occurred in autumn (33.3%), and the lowest in winter (14.5%) (Table 5). Injuries peaked on the weekend, with Sunday (23.9%) and Saturday (17.9%) having the highest incidence (Table 5).

**TABLE 5:** Timing of avulsion (*N* = 117).

	*N* (%)
Season	
Summer	31 (26.5%)
Autumn	39 (33.3%)
Winter	17 (14.5%)
Spring	30 (25.6%)
Day of the week	
Monday	13 (11.1%)
Tuesday	13 (11.1%)
Wednesday	18 (15.4%)
Thursday	13 (11.1%)
Friday	11 (9.4%)
Saturday	21 (17.9%)
Sunday	28 (23.9%)

## DISCUSSION

The demographics of this study population are consistent with the currently published literature on dental traumatology. Paediatric patients accounted for the largest cohort in the study, with a median age of 12 years at the time of the injury. This finding is relatively comparable to recent publications in the literature, with Müller et al. [17] observing a mean of 13.8 years (range 5–82), while Coste et al. [19] observed a mean of 13.3 years (range 5–73). Age has been identified in the literature as a well‐known risk factor for dental trauma [2]. The majority of TDIs occur during childhood and adolescence, with an estimated 71%–92% of all TDIs occurring before 19 years of age [2]. Children have more loosely structured PDL and less mineralised bone reducing resistance to force [6]. However, an increase in older people remaining dentate may result in a shift in this finding, particularly given the elderly are prone to accidental falls. In this cohort, 19.7% of avulsions occurred in people aged 19 and over, and 3.4% in people aged 60 and over. The oldest person included in the study was 90 years of age. Males were observed to experience 2.2 times the frequency of avulsion injuries than females in this study. A meta‐analysis supports this finding with a higher frequency of TDIs observed in males compared to females [20]. Lam [1] reported a closing disparity between TDI prevalence in males and females, likely reflecting an increase in participation in organised sport by females. Such a finding was not reflected in this study.

The median number of visits in the first 12 months was seven visits, although one patient attended the department 20 times. This incurs significant loss of school time and/or work, in addition to the physiologic and psychologic burden of dental trauma. Immature and mature teeth had comparable attendance patterns. Nguyen et al. [21] observed an average treatment time of 7.2 h in the first year of trauma, with almost all children and adults reporting loss of school and work time.

A significant medical history was observed in a minority of patients. However, several older patients with comorbidities and children with complex medical background such as seizure disorders and cerebral palsy were included in the study. Inclusion of such patients may result in patients at higher risk of repeat trauma. Cerebral palsy has been observed to have a high correlation with dental trauma. Holan et al. [22] conducted an observational study at a special needs school in Israel, observing 57% of the 68 included children had signs of trauma to their permanent dentition. People with cerebral palsy have significantly increased frequency of Class II malocclusion [23]. In addition, they have difficulties ambulating and increased prevalence of seizures [22]. This combination of risk factors results in people with cerebral palsy having increased prevalence of dental trauma [23]. Epilepsy has also been identified as increasing risk of dental trauma. Moreira Falci et al. [24] in a systematic review and meta‐analysis observed over five times increased risk of oral and maxillofacial injuries in epileptic patients when compared to healthy individuals (OR 5.22, 95% CI: 2.84–9.36).

Three patients with ADHD were noted in the cohort; however, with an estimated prevalence of 8% of Australian children having ADHD, this appears to be representative of the normal population [25]. Wadia [26] conducted a systematic review and meta‐analysis on the prevalence of dental trauma in children and adolescents with ADHD. They observed an increased prevalence of dental trauma in this cohort compared to their non‐ADHD peers; however, the level of evidence was deemed very low and a causal relationship could not be established.

Fights and sporting injuries are commonly reported causes of tooth avulsion, although significant variation depending on culture and region of the world is noted [4, 8]. Non‐organised sport had the highest prevalence in this study, accounting for 42.7% of the avulsions. Falls and collisions were the second most prevalent aetiology (30.8%). Assaults (12.0%) and organised sport (11.1%) accounted for a minority of injuries. These findings may reflect the large paediatric cohort in this study, where violence was less frequently noted as a cause of injury.

Three injuries included in the study involved trampolines. Trampolines account for 1737 hospitalised injuries nationally annually in Australia between 2002 and 2011, with injury frequency and rate increasing [27]. Injury rates are the highest in children aged 5–9 years of age, correlating with peak age for dental trauma [2]. Trampoline parks have also increased in popularity in recent years in Australia and have been associated with a concerning number of serious injuries [28].

Push bike riding was the most common non‐organised sport resulting in dental avulsion in this study, accounting for 25% of total injuries (*N* = 31). Cycling is a common activity in Australia with 21.4% of Australians reported to cycle at least once a month [29]. Despite a multitude of health benefits, cycling carries risk of traumatic injuries. Helmet use for cycling in Australia is mandatory. Numerous studies have supported the use of helmets in reducing traumatic brain injuries [30]. However, traditional helmets offer no protection to the lower face. Despite this, a recent systematic review Stassen et al. [31] observed a reduced incidence of maxillofacial injury in patients wearing helmets in push bike accidents (OR 0.68). Such findings are supportive of the strict helmet legislation observed in Australia and the high prevalence of dental trauma while cycling. Full‐face helmets have shown to offer additional protection against facial injuries in motorised motorcycle accidents, although there is a paucity of literature on their use in cycling [32, 33]. Full‐face shields have been demonstrated to reduce dental injuries in ice hockey [34]. The IADT supports mandatory wearing of full‐face shields for high risk sports, in conjunction with use of a mouthguard [35]. Faces shields should also be considered for all patients who have sustained a previous significant dental trauma [35].

In addition, scooter injuries featured frequently in our cohort (4.3%). A previous Australian study observed injuries resulting in hospital presentation from scooters include fractures (31.3%) and head injuries (21.8%) [36]. Electric scooters have become increasingly popular in Australia with emerging research observing frequent injury [37]. However, electric scooters remain illegal for use in New South Wales, Australia, where this research was performed.

Two patients included in this cohort were recorded as suffering multiple dental trauma episodes. The first had a historic uncomplicated crown fracture and was included. The second patient had a repeat avulsion injury to the same tooth (with the second injury excluded from the data set). Multiple dental trauma episodes to a tooth have been reported between 8% and 45% of injured teeth [38, 39]. Repeated trauma may reflect oral predisposing factors such as increased overjet and lip incompetence or environmental factors such as risk‐taking behaviours and physical limitations [4].

Concomitant injuries were frequently observed in this cohort. Extra‐oral injuries such as facial lacerations and bony fractures were observed in 43.6% of patients. However, 70.1% of patients were referred for management from an emergency department, which may reflect the very high rate of concomitant injuries. Maxillary central incisors were the most frequently avulsed tooth with no side predilection, as reported in the literature [8].

Sydney has a humid sub‐tropical climate, with mild winters and warm summers, encouraging outdoor recreational activities. Contact sports such as Australian rules football, rugby union and rugby league are winter sports in Australia. The finding of low rates of dental avulsions in winter correlates with the low rates of injuries resulting from organised sports. This may be due to the use of mouthguards in organised sport. The use of mouthguards in organised sport significantly reduces prevalence of TDIs [40]. Mouthguards reduce stress and absorb energy generated by impact to the teeth during sport, thus minimising injury to the teeth and supportive structures [41]. The low rate of dental trauma in organised sport may reflect a high use of mouthguards. Use of appropriate protective equipment in sport, safe public spaces, school policies against bullying and violence and alcohol and drug policies may all additionally assist in reducing dental trauma [4]. This area would benefit from further research. Dental avulsion injury frequency was the highest on the weekend (Sunday 23.9%, and Saturday 17.9%), with the lowest frequency on Friday (9.4%). These data suggest schools and workplaces may be protective against dental injuries compared to leisure time.

There is a lack of consensus in the literature whether TDIs are preventable. This reflects one school of thought that TDIs are unavoidable accidents [4]. However, the literature has demonstrated modifiable risk factors for TDI if addressed, may reduce risk of injury [42]. Oral predisposing factors such as increased overjet of maxillary teeth and lip incompetence increase risk of trauma [5]. Interceptive orthodontics to reduce increased overjet of children in the mixed dentition may decrease risk of trauma; however, the current evidence is heterogenous and at high risk of bias [42]. Nonetheless, interceptive orthodontics may significantly increase treatment burden for children, with longer treatment time and more appointments [42]. However, this also needs to be weighed against the risk of future dental trauma complicating future orthodontic treatment [43]. Oral predisposing factors were excluded in this study due to inconsistencies in record keeping. Increased overjet and lip incompetence were highlighted in multiple cases but patient records did not include sufficient detail of the occlusion. Dental professionals should provide anticipatory guidance to individuals at an elevated risk of dental trauma, including recommending immediate replantation in event of permanent tooth avulsion.

This study was retrospective in origin, presenting a major limitation. Missing data and lack of standardisation were frequently encountered issues. Irregularities between different dental departments was observed in follow up protocol. The paediatric patients' were able to be follow up all patients with a Medicare card (universal health care insurance card in Australia) until 18 years of age. However, adults required a government issued concession card (Health Care Card or Pension Concession Card) for continuing ongoing care after the initial emergency management at WCOH. This resulted in less adult patients' having follow‐up care and inclusion in this study. An additional limitation in sampling was the significant cohort of paediatric patients that had initial management at CHW. CHW is a separate children's hospital network and was not included in this study. Children referred from CHW to WCOH for ongoing care from July 2018 were included, as were children identified in recall clinics as having had a historic trauma. This may have led to selection bias and an underrepresentation of paediatric patients in our cohort, particularly those with more severe injuries. Additionally, record keeping was hand‐written and not standardised. The use of proformas for TDIs and electronic records may assist data management and future research projects.

The inclusion criteria in this study was broader than recent publications on avulsion injuries, including teeth with concomitant crown fractures and root fractures, as well as patients with a significant medical history. Despite the limitations of this retrospective analysis, it provides insight into ‘real world’ management of avulsion injuries in a major tertiary hospital in Australia.

## CONCLUSION

The causes of dental trauma in this study were shown to be varied. Non‐organised sport was the most common aetiology of dental avulsion, which included activities such as scooters, skateboards, push bikes, trampolines and swimming pools. Injuries were most prevalent in the paediatric population and occurred more frequently on weekends. In light of the high frequency of avulsion injuries occurring during non‐organised sport, public health programmes and clinicians should target prevention and anticipatory guidance towards this cohort. Examples include the regulation regarding the use of helmets and mouthguards. Staff working in high‐risk environments should be educated on first aid for dental trauma. The demographics of this cohort reflect regional factors such as weather and high participation in non‐organised and organised sport. Standardisation of records, such as by using a proforma, would assist in future assessment of epidemiological and aetiological parameters of dental trauma.

## CONFLICT OF INTEREST STATEMENT

The authors have no conflict of interest to declare.
